# RAD52: Viral Friend or Foe?

**DOI:** 10.3390/cancers12020399

**Published:** 2020-02-08

**Authors:** Eric A. Hendrickson

**Affiliations:** Department of Biochemistry, Molecular Biology and Biophysics, University of Minnesota Medical School, 6-155 Jackson Hall, 321 Church St., SE., Minneapolis, MN 55455, USA; hendr064@umn.edu

**Keywords:** RAD52, break-induced replication, BIR, single-strand annealing, SSA, alternative non-homologous end joining, A-NHEJ, adeno-associated virus, AAV, human immunodeficiency virus, HIV, herpes simplex virus, HSV

## Abstract

Mammalian Radiation Sensitive 52 (*RAD52*) is a gene whose scientific reputation has recently seen a strong resurgence. In the past decade, RAD52, which was thought to be dispensable for most DNA repair and recombination reactions in mammals, has been shown to be important for a bevy of DNA metabolic pathways. One of these processes is termed break-induced replication (BIR), a mechanism that can be used to re-start broken replication forks and to elongate the ends of chromosomes in telomerase-negative cells. Viruses have historically evolved a myriad of mechanisms in which they either conscript cellular factors or, more frequently, inactivate them as a means to enable their own replication and survival. Recent data suggests that Adeno-Associated Virus (AAV) may replicate its DNA in a BIR-like fashion and/or utilize RAD52 to facilitate viral transduction and, as such, likely conscripts/requires the host RAD52 protein to promote its perpetuation.

## 1. Pathways of DNA Double-Stranded Break Repair

**Figure 1:**
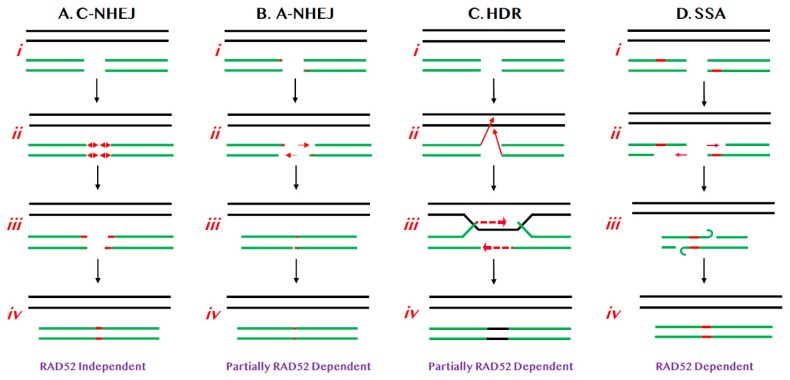
Relevant pathways of DNA DSB repair for this review. (**A**) C-NHEJ. (i) Chromosomes are diagrammed as double lines. A DSB is diagrammed as occurring in either a sister chromatid or a homologue (broken green lines). (ii) Limited processing (red lines with double arrowheads) results in (iii) the insertion or deletion of small numbers of nts (red lines). (iv) DNA LIGIV subsequently seals the DSB. (**B**) A-NHEJ. (i) As above. (ii) Limited 5′ > 3′ resection (red arrows) occurs, exposing regions of microhomology (red bars). (iii) These regions of microhomology are used to re-align the chromosomal ends and (iv) then the DSB is religated. (**C**) HDR. (i) As above. (ii) The 3′-ends of the DSB invade (red arrows) the undamaged sister chromatid or homologue. (iii) Limited DNA synthesis (dashed red arrows) then reconstitutes the missing genetic information and (iv) the DSB is religated such that the new information is identical to the donor chromatid/chromosome (black line). (**D**) SSA. (i) As above. (ii) Limited 5′ > 3′ resection (red arrows) occurs, exposing regions of homology (red bars). (iii) These regions of homology are used to re-align the chromosomal ends and (iv) then the DSB is religated.

DNA double-stranded breaks (DSBs) are the most toxic form of genomic lesions for human cells. These lesions can arise from both endogenous DNA replication errors and via exogenous exposure to agents that damage DNA. In yeast, the toxicity of DSBs is so profound that even a single unrepaired DSB can lead to cell death [1]. To ensure that DSBs are repaired, mammals have developed at least two major pathways of DSB repair—classic non-homologous end joining (C-NHEJ; Figure 1A) [2] and homology-directed repair (HDR; Figure 1C) [3]—that are differentially utilized depending upon the actual DSB lesion, the stage of the cell cycle and the organism in which the lesion occurs [4,5]. DSB repair *in toto* is an exceptionally complicated process that requires literally hundreds of different factors in order to be accomplished successfully. One of these factors, RAD52, will be the focus of this review.

### 1.1. NHEJ-Mediated DSB Repair

C-NHEJ is a process that results in the covalent ligation of the two broken ends of a DSB in the most expedient (if not always the most accurate) way possible, in an attempt to restore the physical integrity of the affected chromosome (Figure 1A). The mechanism of C-NHEJ is very well understood and the pathway is dependent upon a heterodimeric protein called Ku, a number of accessory factors, as well as a dedicated DNA ligase (DNA Ligase IV or LIGIV) [6]. C-NHEJ is preferentially utilized during G1 of the cell cycle [7] and it is thought to be predominately, but not exclusively [8], an error-prone process that frequently results in the generation of small (two to three nucleotides; nts) insertions or deletions (aka “indels”) at the site of repair (Figure 1A(ii,iii)) [2]. Superficially, one might not predict that an error-prone process would even exist, let alone be evolutionarily preferred, but, counter-intuitively, C-NHEJ is indeed the major pathway of DSB repair for human cells. Ironically, tumor cells that are defective for HDR (see below) are forced to rely solely on mutagenic NHEJ pathways to maintain genome integrity and, because of this, these cells now show increased sensitivity to chemo [9,10] and synthetic-lethality-based therapies [11,12,13]. Importantly, there is no evidence to suggest that RAD52 plays any role in C-NHEJ.

Alternative-NHEJ (A-NHEJ) is a more recently described pathway [14] where the mechanism is less well understood (Figure 1B). In A-NHEJ, short resection, possibly mediated by the MRE11:RAD50:NBS1 (MRN) complex, occurs at the ends of the DSB (Figure 1B(ii)). If this resection exposes regions of “microhomology”—typically equal to or greater than 3 nts [15]—these can be used to reanneal and eventually repair the ends (Figure 1B(iii,iv)). Because of this reaction mechanism, A-NHEJ perforce always causes small deletions. A-NHEJ is partially dependent upon RAD52 [16], possibly due to the requirement for strand annealing, which is RAD52’s seminal activity [12,13,17].

### 1.2. HDR-Mediated DSB Repair

HDR precisely repairs DSBs using the genetic information provided from a homology donor predominately in S phase of the cell cycle (Figure 1C). In particular, HDR is required to repair complex genomic lesions such as inter-strand crosslinks and stalled replication forks. Besides DNA repair and replication, HDR also plays essential roles in meiotic recombination [18] and telomere maintenance [19]. Furthermore, deficits in HDR, caused by mutations in Breast Cancer Allele 1 or 2 (BRCA1 or BRCA2), predispose patients to breast and ovarian cancers [20,21,22]. Interestingly, while the main HDR pathway is not dependent on RAD52, it is nonetheless attenuated in its absence (Figure 1C) [16].

HDR is initiated by strand invasion events following by limited DNA synthesis (Figure 1C) and, from a purely functional point of view, replaces the genetic information lost at the site of the DSB with the identical information from the donor sister chromatid/chromosome resulting in quasi error-free repair. HDR is composed of many subpathways in addition to the canonical HDR pathway (Figure 1C), but only one, single strand annealing (SSA; Figure 1D) [23,24] will be highlighted here. SSA is unique amongst HDR reactions in that it is not accompanied by a strand invasion event, but requires end resection (again, likely mediated by the MRN complex) followed by the annealing of repetitive elements respectively located on opposing strands (Figure 1D) [25,26,27,28,29], thus bridging the ends of the DSB. Conceptually, SSA is indistinguishable from A-NHEJ (compare Figure 1D with Figure 1B) and it is similar to A-NHEJ (and very unusual for an HDR reaction) error-prone as it is always accompanied by deletions. The difference between the two pathways is solely in the size of homology required for the reactions to proceed: in the case of A-NHEJ the homology requirement is ~3 nts whereas much longer stretches of homology (>30 nts; [30]) are required for SSA. Which pathway of DSB repair is engaged for a particular repair event is determined by a variety of factors including, but not limited to, the cell type in which the DSB occurs, the stage of the cell cycle when the DSB occurs or is repaired, the proximity of repetitive sequences to the DSB, and the expression levels of the relevant DNA repair factors. Regardless, RAD52 contributes to A-NHEJ, all of the HDR subpathways and is absolutely required for SSA (Figure 1D).

## 2. RAD52 and BIR

RAD52 forms a heptameric ring whose primary activity is to bind double-stranded DNA (dsDNA). In yeast, *RAD52* is arguably the most important DNA repair gene and its activities are required for almost all fungal repair and recombination reactions [31]. Early on, a large number of loss-of-function studies suggested that RAD52’s role (s) had been diminished in higher eukaryotes [32,33,34]. In the past decade, however, research on RAD52 has been buoyed and reenergized by studies implicating RAD52 in a wide variety of DNA metabolic reactions. Due to RAD52’s popularity, the reader is directed to any number of recent, comprehensive reviews for specifics on RAD52’s abundant purported activities [12,13,17]. For the sake of this review, we will focus predominately on one of these activities: BIR.

**Figure 2:**
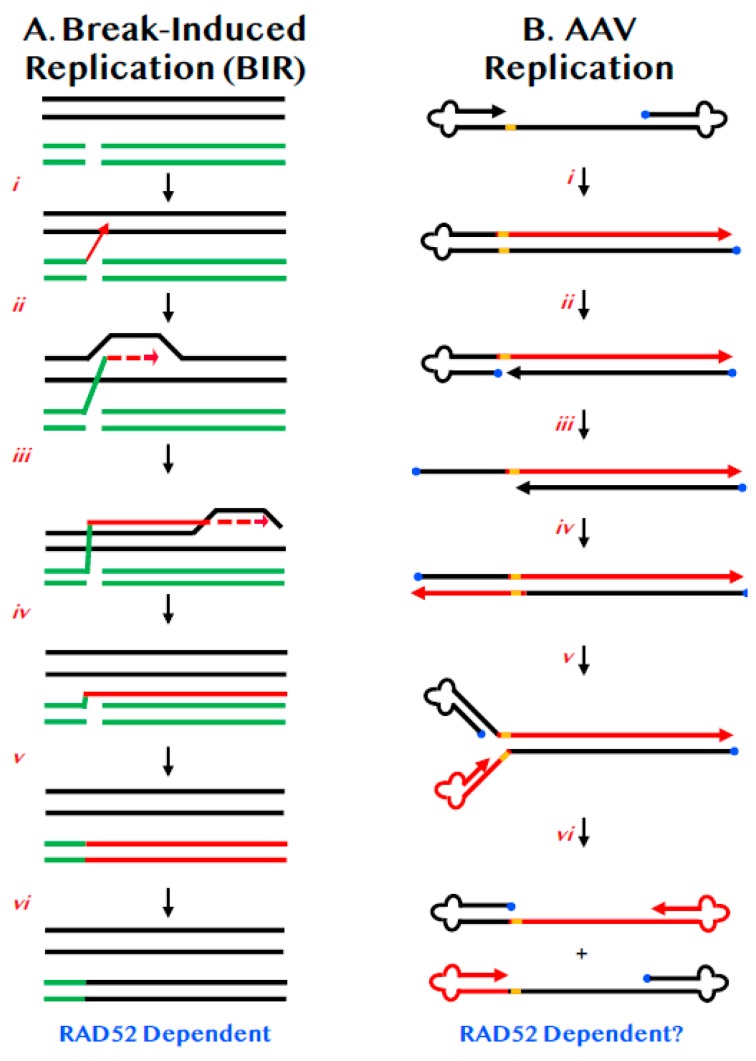
BIR and its similarities to AAV replication. (**A**) Chromosomes are diagrammed as double lines. A DSB is diagrammed as occurring in either a sister chromatid or a homologue (broken green lines). (i) One of the 3′-ends of the DSB invades (red arrow) the undamaged sister chromatid or homologue. (ii) Limited DNA synthesis begins (dashed red arrow). (iii) DNA synthesis continues along the chromosome by displacement synthesis (solid and dashed red lines). (iv) DNA synthesis can continue all the way to the end of the chromosome (red line). (v) The complementary strand is then replicated (double red line). (vi) Repair is complete with the repaired chromosome now possessing DNA sequences identical to the donor (black lines). (**B**) A diagram of AAV DNA is shown. The 3′-end is denoted by the arrowhead. The 5′-end is occupied by the Rep protein (blue circle). The viral ITRs are shown as cloverleafs and the trs as an orange bar. (i) DNA synthesis (red line) can proceed in a unidirectional manner from the 3′-OH to the end of the viral DNA. (ii) Rep protein binds and nicks at the trs site. (iii) The left-most ITR is shown as unfolding but whether this requires concomitant DNA synthesis is unclear. (iv) DNA synthesis (red line) proceeds in a conservative, unidirectional manner to the viral end. (v) The left-most ITRs are shown re-annealing. (vi) The right-most ITRs are shown re-annealing.

Normal chromosomal DNA replication begins at origins and precedes bi-directionally in a semi-discontinuous and semi-conservative manner. BIR is a highly specialized version of HDR that differs from canonical replication in that it involves only one end of DNA, precedes via uni-directional bubble migration and generates progeny DNA in a completely conservative fashion (Figure 2A) [35,36]. Like most HDR events, BIR begins with a strand invasion event (Figure 2A(ii)). Unlike HDR, which generally only involve small regions of DNA synthesis (Figure 1C(iii)), in BIR, the invading strand assembles the equivalent of a functional replication fork and then engages in extensive DNA replication (Figure 2A(iii,iv)). Indeed, when BIR is associated with repair near telomeres the BIR event may proceed completely to the end of the chromosome (Figure 2A(v)). The DNA strand generated by such a BIR event can be converted to dsDNA via semi-discontinuous DNA replication using a mechanism that involves primarily DNA polymerase δ (Figure 2A(v)) [37]. This mechanism ensures that the DSB is repaired, but in so doing, generates only conservative products of DNA replication (Figure 2A(vi)). BIR is mechanistically likely not the pathway of choice for normal DNA DSB repair as it is associated with loss-of-heterozygosity, high mutation rates [38] and enhanced tri-nucleotide expansions [39]. As a consequence, it has probably been evolutionarily retained more as a last-gasp chromosome salvage pathway. Importantly however, and although the mechanism is still quite undefined, RAD52 is required for BIR in both yeast [40] and humans [41].

## 3. AAV Replication and Integration

Viruses are parasites and as such they are either restricted by or depend upon the host cells they infect. In many instances, the host has evolved adequate defense systems against viral infection and so the virus needs to inactivate these defense systems in order to mount a productive infection. Examples of this would include the expression of the viral infectivity factor (VIF) protein of certain retroviruses that inactivates the cellular apolipoprotein B mRNA editing enzyme, catalytic polypeptide-like (APOBEC) family of proteins that would otherwise lethally mutagenize the viral cDNA [42]. Similarly, adenoviruses uniformly inactivate (often by targeted proteolysis) specific cellular replication and repair factors (e.g., MRN) such that the viral replication can proceed unimpeded [43]. There are also examples where viruses must conscript cellular factors to enable their life cycle. Below, I discuss the (albeit limited) evidence that AAV may replicate its DNA in a BIR-like fashion and/or require endogenous RAD52 at later stages of the infection to be viable.

AAV is a human parvovirus that has both latent and lytic lifecycles [44,45]. The ability to undergo a latent infection is a necessary part of the normal AAV life cycle since AAV requires the presence of a helper virus (generally adenovirus; AdV) for a productive lytic infection. Thus in the general population, lytic AAV has only been detected in individuals undergoing the symptoms of an AdV [46,47]) or herpes simplex virus (HSV; [47]) infection. Approximately 85% of the human population is seropositive for AAV, although there is no pathology nor disease that has been attributed to an AAV infection [47].

For a brief while, AAV’s greatest use was as a gene editing tool in human cells [45,48], however that use has recently been completely superseded by the Clustered Regularly Interspaced Short Palindromic Repeats (CRISPR) technology. As a gene delivery tool, however, AAV continues to be exceptionally popular with genome engineers. This popularity stems from many factors including ease of use, but is highlighted by its inability to spread to other non-target tissues without a helper virus and by a large number of serotypes that permit the design of AAV-based gene delivery vectors to many mammalian organs with good degrees of specificity (see, for example: [49,50]).

AAV is a single-stranded DNA virus of 4.68 kb. Either strand of the integrated AAV DNA can be packaged [51] and both are equally infectious [52]. AAV is so small that it only encodes two genes; only one of which, replication (Rep) is required for viral replication [44]. These genes are flanked by identical inverted terminal repeats (ITRs) of 146 nts that form T-shaped hairpins, due to three palindromic sequences residing within the ITRs [53]. The ITRs are the only *cis*-acting sequences necessary for viral replication [54], which enhanced its development as a vector for gene delivery. The replication of AAV is relatively well-understood. The free 3′-OH on the viral single-strand is used by cellular polymerases to extend that strand all the way to the end of the virus, duplicating one of the ITRs (Figure 2B(i)). The Rep protein then binds and nicks the terminal resolution site (trs) on the parental strand—and in so doing becomes covalently linked to the 5′-end—which generates a new 3′-OH end (Figure 2B(ii)). This intermediate is then extended by DNA synthesis through the remaining ITR (Figure 2B(iii,iv)). When the ITRs reform, they generate single-stranded viral DNA of either polarity (Figure 2B(v,vi)).

The viral DNA produced by this replication has two potential fates. It can be re-replicated, generating even more progeny for a lytic cycle. Alternatively, it can stably integrate into the human genome, preferentially, but not exclusively, at a site on chromosome 19 [55,56]. If a recombinant form of the virus has been used that contains portions of human genomic DNA, then that virus is also capable of undergoing HDR-dependent gene editing events [45,57].

## 4. AAV Replication, Integration and RAD52

### 4.1. AAV Replication and RAD52

Inherent to all of these viral reactions described above is the simple fact that AAV is so small that it encodes for only two of its own proteins. Thus, perforce, it is almost inconceivable that AAV does not utilize some endogenous cellular factors for the various tasks of DNA replication and cellular transduction. Surprisingly, given the importance and heavy usage of AAV, what these factors are, however, is much less well defined. Two studies have investigated the absence of RAD52 on viral DNA integration, one carried out in murine cells [58] and one in human cells [16]. In both studies it was reported that the frequency of random AAV integration was significantly reduced in the absence of RAD52; strongly implying that RAD52 is normally a pro-viral factor. In neither study, however, was the status of viral DNA replication accurately assessed. This was unfortunate because many aspects of AAV replication resemble BIR, a RAD52-dependent process. Thus, the resulting progeny viral DNAs are neither all parental nor all newly synthesized strands, but consist of portions of each (Figure 2B(vi)). Most importantly perhaps, viral DNA synthesis proceeds in a simple, unidirectional manner highly reminiscent of BIR (compare Figure 2B with Figure 2A). Lastly, the reformation of the viral ITRs requires strand annealing (in this instance, intramolecularly) that is likely to be facilitated by RAD52. The fact that RAD52 can be chromatin immunoprecipitated from AAV ITRs [58] is consistent with this possibility. Thus, it is tempting to postulate that RAD52 may be required for AAV DNA replication. If this were the case, then the decrease in viral integration could simply be an indirect effect due to a reduced amount of viral DNA.

### 4.2. AAV Integration and RAD52

**Figure 3:**
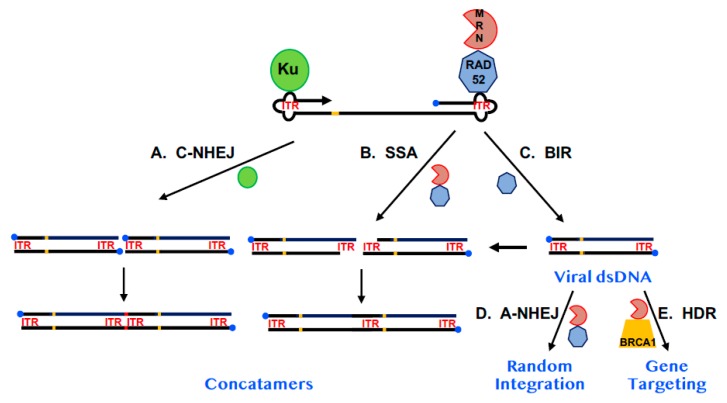
A model for the impact of RAD52 on AAV replication and integration. A diagram of AAV DNA is shown. The 3′-end is denoted by the arrowhead. The 5′-end is occupied by the Rep protein (blue circle). The viral ITRs are shown as cloverleafs and designated as ITR in red and the trs is shown as an orange bar. The Ku protein is diagrammed as a green circle, RAD52 as a heptagon and the MRN complex as a PacMan-like figure. (**A**) The impact of C-NHEJ. When Ku binds to AAV and engages C-NHEJ, concatemerization of the viral dsDNA (which may require replication of viral DNA—see (**C**) on the far right) occurs resulting in the loss of the Rep protein from internal sites and the formation of small indels (red bar) at the site of fusion. (**B**) The impact of SSA. MRN may process the viral dsDNA (which may require replication of viral DNA—see (**C**) on the far right), leaving 3-end overhangs consisting of repetitive ITR sequences. These overhangs can be used in a RAD52-dependent SSA reaction to generate concatemers in which one of the two annealing ITRs has been deleted. (**C**) The impact of BIR. As depicted in Figure 2, RAD52 may facilitate, via a BIR-like mechanism, the replication of single-stranded AAV DNA into dsDNA. (**D**) The impact of A-NHEJ. Some of the viral dsDNA may, in a RAD52- and A-NHEJ-dependent fashion, randomly integrate into the host genome, establishing AAV as a lysogen. MRN is likely also involved in this pathway. (**E**) The impact of HDR. If the AAV is a recombinant vector and contains sequences homologous to a human chromosome it has the additional possibility of undergoing a gene targeting event at the site of chromosomal homology. This pathway is likely dependent upon MRN and BRCA1 and, to a lesser degree, RAD52.

Arguing against the hypothesis that RAD52 plays a significant role in AAV replication was the observation that while random viral integration was reduced in RAD52-null cells [16,58], the occurrence of gene-targeted events was virtually unaffected [16]. It is difficult to envision a mechanism where a simple reduction in viral DNA would give rise to fewer random integration events, but not to targeted ones. Instead, it seems plausible that even if RAD52 does play a role in viral DNA replication, its absence is most important at later steps in the transduction process. A likely, albeit hypothetical, model for AAV replication and transduction postulates that much of the viral fate is regulated by which cellular proteins bind to the viral ITRs (Figure 3). As mentioned above, RAD52 binds to the ITRs, perhaps in conjunction with the MRN complex [59] and together these proteins are in competition with the C-NHEJ factor, Ku [58]. The binding of Ku to the ITRs likely activates the C-NHEJ pathway to concatemerize the DNA, which is a likely viral dead end (Figure 3A). Similarly, if RAD52/MRN bind on to the ends, this could lead to processing of the viral DNA by SSA and potentially also concatemerized it (Figure 3B). In this context, RAD52 is performing an antiviral function. However, some of the RAD52 may promote viral DNA replication, potentially through BIR (Figure 3C), which would increase the concentration of viral dsDNA. This DNA would then either randomly integrate in a RAD52/MRN-dependent, A-NHEJ dependent fashion (Figure 3D) or, if the AAV was carrying exogenous sequences homologous to a chromosome it could undergo gene targeting in a BRCA1/MRN-dependent, HDR-dependent fashion (Figure 3E). In its favor, this model nicely explains why the ablation of RAD52 would reduce viral transduction. Thus, any potential increase in viral dsDNA caused by the ablation of SSA and reduced concatemer formation could be offset by a reduction in BIR. Most importantly, whatever the final level of viral dsDNA there is, the absence of RAD52 would preferentially negatively impact A-NHEJ rather than HDR since the former is more dependent upon RAD52 [16] whereas the latter appears more dependent upon BRCA1 [12,13,17]. This model is also consistent with the known mechanism of rAAV random integration. Because the viral ITRs are extensively altered with deletions and there is frequent microhomology at the chromosomal junctions, the insertion of the viral dsDNA replication intermediate into the genome is widely believed to occur through A-NHEJ [60,61].

## 5. Other Viruses and RAD52

The available evidence summarized above is consistent with RAD52 providing AAV with (mostly) pro-viral activities. Unfortunately, what is good for the goose is not always good for the gander and in the case of RAD52, whether it is pro-viral or anti-viral seems to depend strongly on which virus is involved.

### 5.1. Human Immunodeficiency Virus (HIV) and RAD52

HIV is a human retrovirus that has an immense impact on human health and is arguably the most widely-studied virus on the planet [62]. Because of this, the viral life cycle including replication and transduction is well understood. Superficially, HIV looks like a poster child for utilizing RAD52/BIR as its mode of DNA synthesis, since it requires a mandatory annealing reaction between the HIV long terminal repeats (LTRs) and DNA synthesis is unidirectional and conservative. In addition, RAD52 has been shown to bind to the retroviral LTRs [63]. However, when HIV replication was assessed in either murine RAD52 knockout cell lines or human RAD52 knockdown cell lines, HIV transduction was actually extremely elevated [63]; thus, endogenous RAD52 has clear anti-viral activity when it comes to HIV infections. The exact mechanism behind this effect is not known, but the ability of RAD52 to utilize SSA to concatermerize viral dsDNA or to generate circles from the viral double-stranded cDNA (both of which products are likely not productive for an HIV infection) is certainly important [64]. What is most important, however, is that while AAV utilizes the RAD52-influenced A-NHEJ pathway to integrate into the genome, HIV encodes its own (RAD52-independent) integrase [65] and consequently HIV integration is unaffected by the absence of RAD52. Thus, because the mechanisms of viral transduction are different, the absence of RAD52 has a negative effect in the case of AAV and a positive effect in the case of HIV.

### 5.2. Herpes Simplex Virus (HSV) Replication

HSV is a human pathogen that can cause cold sores (HSV-1) or genital lesions (HSV-2) [66]. Although it is utilized less often than AAV, HSV-based gene therapy vectors still enjoy widespread use in clinical and basic research [67]. Like AAV, HSV enjoys both a lytic and a lysogenic lifestyle. Indeed, the ability of lysogenic HSV to reactivate and undergo a lytic infection is what accounts for much of its public health burden and poor reputation. In contrast to AAV and HIV, the presence or absence of RAD52 appears to be largely irrelevant for normal HSV replication and latency [68]. Perhaps not surprisingly, the explanation for this appears to be due to the fact that HSV utilizes yet a different mechanism for replication than either AAV or HIV [69]. Specifically, in the case of HSV, and in contrast to AAV and HIV, there appears to be a positive requirement for an SSA-like recombination function to generate infectious HSV progeny DNA [70]. Ironically, this requirement is apparently so strong that the virus has evolved its own SSA-like protein, Infected Cell Protein 8 (ICP8) [68]. Thus, it is not so much that RAD52 is irrelevant for HSV replication, but rather that the virus expresses a protein with similar functions. Indeed, when a virus lacking ICP8 is utilized to infect human cells, RAD52 can at least partially compensate for the absence of ICP8 [68].

## 6. Conclusions

Viruses are, first and foremost, concerned with the importance of their own propagation. Therefore, they have evolved to either enslave or inactivate host cellular factors to ensure their successful survival. RAD52 is a host protein with strand-annealing activity that appears to play roles in many nucleic acid metabolic pathways [12,13,17]. Not surprisingly, it turns out to be a central target for viral regulation: HIV would prefer to inactivate RAD52 whereas AAV conscripts RAD52 to help with viral integration. HSV is more akin to AAV but has taken matters to an extreme, by evolving a viral protein, ICP8, that has many of RAD52’s activities. Consequently, RAD52 could make an attractive anti-viral target, with the strong caveat that one needs to appreciate whether the virus in question would be better off with enhanced or reduced RAD52 activity. The development of small molecule modulators of RAD52 could therefore likely see utility as antiviral reagents.
